# Supplementary material for: The Impact of Glomerular Disease on Dyslipidemia in Pediatric Patients Treated with Dialysis
Source: Nutrients. 2025 Jan 27;17(3):459. doi: 10.3390/nu17030459 (PMC11819668; doi:10.3390/nu17030459)
Supplement: Supplementary file 1 [file nutrients-17-00459-s001.zip › nutrients-3422443-supplementary.pdf]

Figure S1. Cohort construction based on eligibility criteria.

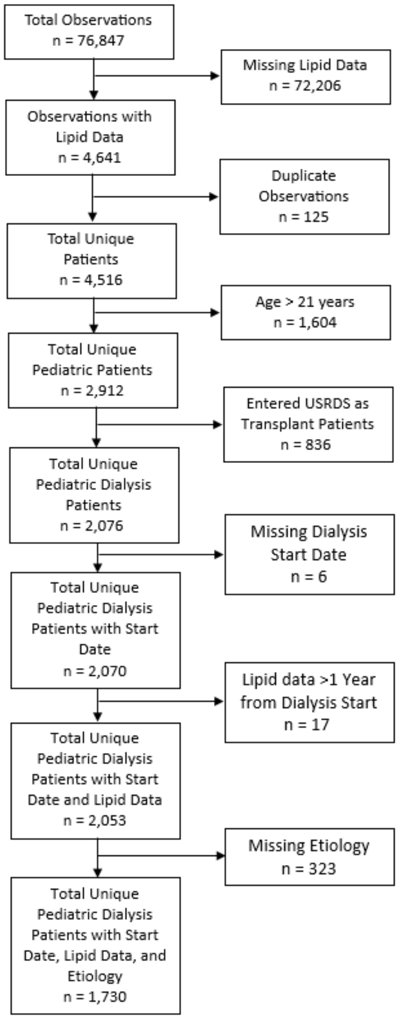

Table S1. National Heart, Lung. Blood Institute (NHLBI) guidelines for lipid parameters based on patient age [10].

Lipid parameter, mg/dL	Acceptable	Borderline	Abnormal
TC	0-19yo: <170	0-19yo: 170-199	0-19yo: ≥200
	20-21yo: <190	20-21yo: 190-224	20-21yo: ≥225
LDL-C	0-19yo: <110	0-19yo: 110-129	0-19yo: ≥130
	20-21yo: <120	20-21yo: 120-159	20-21yo: ≥160
TG	0-9yo: <75	0-9yo: 75-99	0-9yo: ≥100
	10-19yo: <90	10-19yo: 90-129	10-19yo: ≥130
	20-21yo: <115	20-21yo: 115-149	20-21yo: ≥150
HDL-C	0-21yo: >45	0-21yo: 40-45	0-21yo: <40

TC = total cholesterol, LDL-C = low density lipoprotein cholesterol, TG = triglyceride, HDL-C = high density lipoprotein cholesterol.

Table S2. Unadjusted comparison of lipid measures by sex.

Lipid parameter, mg/dL	Age 0-9 years median (IQR)	Age 10-21 years median (IQR)	p-value
n	*280*	*1450*	
TC	185 (145, 235)	171 (137, 210)	0.002
LDL-C	98 (68, 133)	97 (71, 126)	1
TG	206 (132, 290)	153 (107, 223)	<0.0001
HDL-C	41 (31, 52)	38 (30, 48)	0.03

Each lipid parameter is presented as the median value in mg/dL with the interquartile range (IQR) and compared based on sex. TC = total cholesterol, LDL-C = low density lipoprotein cholesterol, TG = triglyceride, HDL-C = high density lipoprotein cholesterol.

Table S3. Unadjusted comparison of lipid measures by age less than 10 years and age greater than 10 years old.

Lipid parameter, mg/dL	Male median (IQR)	Female median (IQR)	p-value
n	*942*	*788*	
TC	168 (134, 211)	179 (142, 226)	<0.0001
LDL-C	95 (69, 129)	103 (73, 136)	<0.0001
TG	157 (110, 235)	161 (109, 241)	<0.0001
HDL-C	36 (29, 47)	40 (31, 52)	<0.0001

Each lipid parameter is presented as the median value in mg/dL with the interquartile range (IQR) and compared based on sex. TC = total cholesterol, LDL-C = low density lipoprotein cholesterol, TG = triglyceride, HDL-C = high density lipoprotein cholesterol.
